# Real-time monitoring of *Ralstonia solanacearum* infection progress in tomato and Arabidopsis using bioluminescence imaging technology

**DOI:** 10.1186/s13007-022-00841-x

**Published:** 2022-01-15

**Authors:** Cuihong Xu, Lingkun Zhong, Zeming Huang, Chenying Li, Jiazhang Lian, Xuefang Zheng, Yan Liang

**Affiliations:** 1 Ministry of Agriculture Key Lab of Molecular Biology of Crop Pathogens and Insects, Institute of Biotechnology, Zhejiang University, Hangzhou, 310058 China; 2 Key Laboratory of Biomass Chemical Engineering of Ministry of Education, College of Chemical and Biological Engineering, Zhejiang University, Hangzhou, 310027 China; 3 Agricultural Bioresources Research Institute, Fujian Academy of Agricultural Sciences, No. 247 Wusi Road, Fuzhou, 350003 China

**Keywords:** *Ralstonia solanacearum*, Arabidopsis, Bioluminescence, *Lux*CDABE, Bacterial wilt

## Abstract

**Background:**

*Ralstonia solanacearum*, one of the most devastating bacterial plant pathogens, is the causal agent of bacterial wilt. Recently, several studies on resistance to bacterial wilt have been conducted using the Arabidopsis-*R. solanacearum* system. However, the progress of *R. solanacearum* infection in Arabidopsis is still unclear.

**Results:**

We generated a bioluminescent *R. solanacearum* by expressing plasmid-based *lux*CDABE. Expression of *lux*CDABE did not alter the bacterial growth and pathogenicity. The light intensity of bioluminescent *R. solanacearum* was linearly related to bacterial concentrations from 10^4^ to 10^8^ CFU·mL^−1^. After root inoculation with bioluminescent *R. solanacearum* strain, light signals in tomato and Arabidopsis were found to be transported from roots to stems via the vasculature. Quantification of light intensity from the bioluminescent strain accurately reported the difference in disease resistance between Arabidopsis wild type and resistant mutants.

**Conclusions:**

Bioluminescent *R. solanacearum* strain spatially and quantitatively measured bacterial growth in tomato and Arabidopsis, and offered a tool for the high-throughput study of *R. solanacearum*-Arabidopsis interaction in the future.

## Background

Bacterial wilt is a soil-borne bacterial disease caused by *Ralstonia solanacearum*, the second most devastating bacterium among plant pathogens. *R. solanacearum* can infect more than 250 plants, including tomato (*Solanum lycopersicum*), potato (*S. tuberosum*), banana (*Musa nana*), and other agriculturally important crops [1, 2]. After infection through wounds, root tips, or cracks in the lateral roots, *R. solanacearum* colonizes the root cortex, invades and multiplies in xylem vessels, and reaches the aerial parts of the plant, causing wilting symptoms and subsequent plant death [3, 4]. Control of bacterial wilt is a challenge because of the lack of available commercial resistant varieties and the long survival time of *R. solanacearum* in soil, water, and infected plant tissues.

Several studies on the molecular mechanism of disease resistance to *R. solanacearum* have been conducted using the Arabidopsis-*R. solanacearum* system owing to the availability of genetic resources and well-defined technology for this model plant species [5–10]. Study of a genetic mapping population from the cross between a susceptible (Col-5) and resistant ecotype (Nd-1) revealed that an atypical resistance gene (*R* gene) encoding RRS1 (resistance to *Ralstonia solanacearum* 1) confered resistance against *R. solanacearum* strain GMI1000 [5]. Interestingly, mutation in a neighboring gene encoding a well-known R protein (RPS4, resistance to *Pseudomonas syringae* 4) also impaired resistance to *R. solanacearum* [11]. Recently, using the Arabidopsis-*R. solanacearum* system, many regulatory genes, including suppressor of g2 allele of skp1 (*SGT1*), glutamate decarboxylases (*GAD*s), LRR receptor-like kinases, such as *ERECTA*, *CLAVATA1*, and *CLAVATA2*, were found to function in resistance to *R. solanacearum* [7, 9, 10]. In addition, the involvement of phytohormone ethylene, salicylic acid (SA) and jasmonic acid signaling pathways in resistance to *R. solanacearum* was also studied using the Arabidopsis-*R. solanacearum* system [7, 12, 13]. Although these studies were conducted in Arabidopsis, the spatial distribution and infection progress of *R. solanacearum* in Arabidopsis is still unclear.

To guarantee the success of long-term breeding programs, it is crucial to develop a disease detection approach that allows easy tracking and quantification of bacterial colonization. A few methods are available for the detection of *R. solanacearum*, such as disease index, colony counting, and DNA-based amplification assay using polymerase chain reaction (PCR). Disease index is estimated by scoring the wilting severity; therefore, there is a possibility of human error in the evaluation of resistant varieties [14, 15]. The colony counting method can be relatively accurate in quantifying bacteria, but it is time-consuming and labor-intensive, and the sample heterogeneity in different location of tissues might cause errors as well [14, 15]. PCR assays are relatively sensitive to detect diseases, particularly diseases at an early stage; however, this method usually requires either expensive thermocycling equipment or expensive chemicals, and the laboratory skills to perform the technical procedures and data analysis [16]. In addition, none of these methods can systematically and comprehensively reflect the incidence of disease in the whole plant.

In recent years, bioluminescence imaging technology, mostly developed based on the expression of *lux*CDABE encoding autonomous light-emitting elements, has been successfully applied to engineer plant pathogenic bacteria [17, 18]. The genes *lux*A and *lux*B encode the heterodimeric enzyme luciferase, while *lux*C, *lux*D, and *lux*E are responsible for the synthesis of fatty aldehydes that serve as substrates for the luminescence reaction of luciferase, such that, the cells expressing *lux*CDABE operon will emit light autonomously [19]. The emitted light can be captured using a sensitive detector that converts light signals into electrical values, which are then digitalized and displayed on a computer monitor instantaneously. As the light signal produced by *lux*CDABE has a long life, high sensitivity, and strong specificity, the plant pathogens harboring *lux*CDABE can be directly monitored without destroying plant tissues after inoculation [20–22]. In addition, since the expression of *lux*CDABE is linearly correlated with the concentrations of living bacteria, the relative light intensity can be used to quickly quantify the levels of bacteria [23, 24]. Therefore, bioluminescent imaging technology provides a non-invasive method to study the spatial distribution and infection progress of pathogens, and quickly score the disease severity.

In this study, we generated a bioluminescent *R. solanacearum* strain (FJ91-LUX) by transforming a plasmid containing the *lux*CDABE operon into *R. solanacearum* FJ91, a strain isolated from Fujian, China. Transformation of the *lux*CDABE operon had no significant impact on *R. solanacearum* growth and pathogenicity in tomato and Arabidopsis. Light emission from the FJ91-LUX strain was detectable when concentration of the strain was above 10^4^ CFU·mL^−1^ and was linearly related to bacterial concentrations below 10^8^ CFU·mL^−1^. We monitored the infection progress in tomato and Arabidopsis after root inoculation with the FJ91-LUX strain and found that light signals were transmitted from roots to stems within 3 days and were mainly observed in the stems. We also compared the light intensity between Arabidopsis wild type and mutants defective in ethylene and SA signaling pathways after inoculation with FJ91-LUX, and it was found that the light intensity in mutants were significantly different from that in wild type. Thus, bioluminescent *R. solanacearum* can be used to study the defense mechanism and to facilitate isolation of disease-resistant varieties in the future.

## Results

### Generation of a bioluminescent *R. solanacearum* strain

**Fig. 1:**
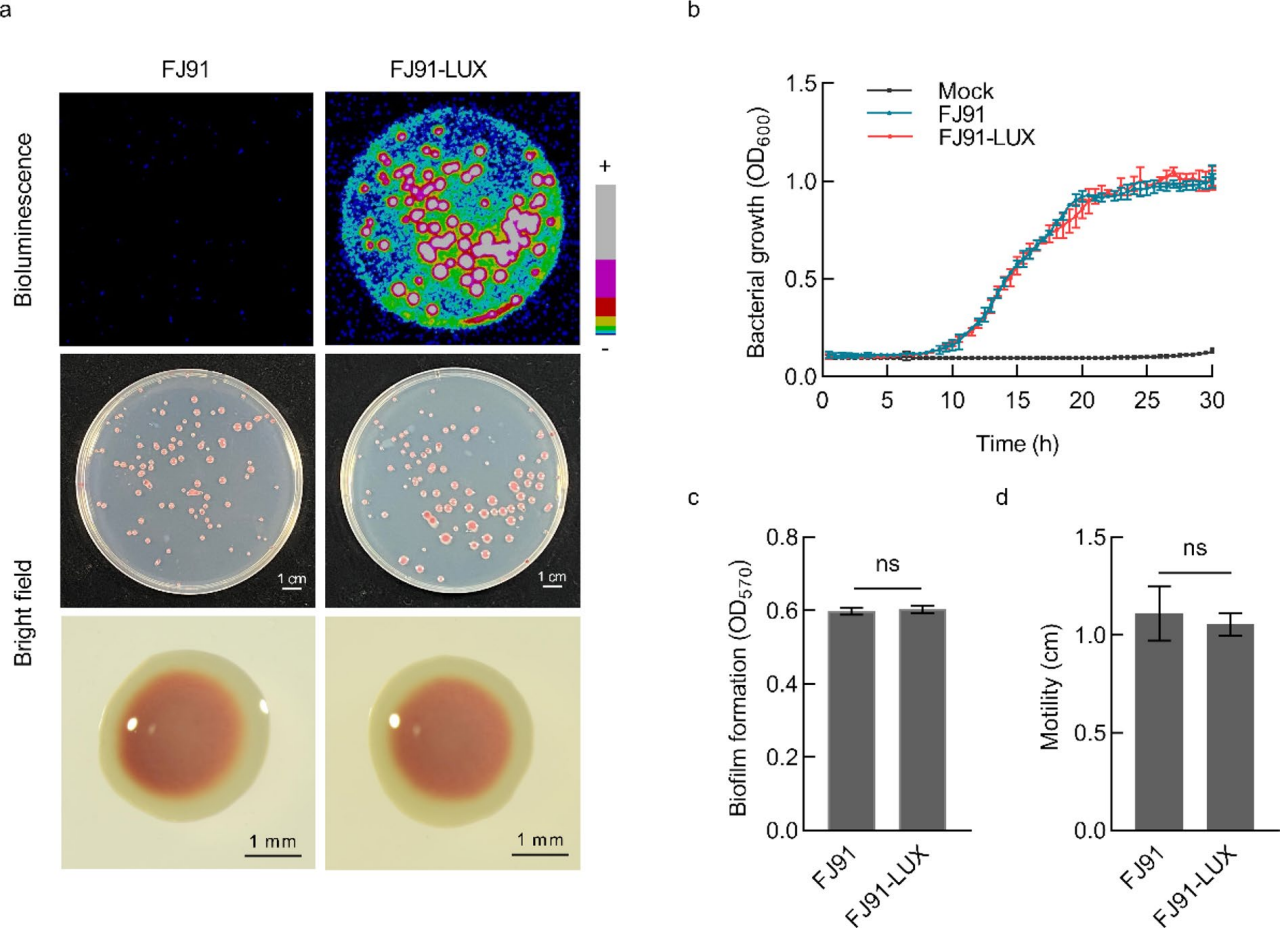
Bacterial characteristics of *Ralstonia solanacearum* FJ91-LUX are not significantly different from those of FJ91. **a** Bacterial morphology and bioluminescence signals. Bioluminescent signals were detected using a photon-camera. + : Strong; −: Weak. **b** Bacterial growth curves. Bacterial growth was determined by measuring the optical density of media (OD_600_). Data are shown as the mean ± SD (n = 3). **c** Biofilm biomass. The amount of biofilm formed was quantified using crystal violet adherence assay. Data are shown as the mean ± SD (n = 4), and ns indicates no significant difference between FJ91 and FJ91-LUX (*t*-test). **d** Bacterial motility. Surface motility was evaluated by measuring the colony diameter of bacterial growth 48 h after inoculating the center of the plates. Data are shown as the mean ± SD (n = 4), and ns indicates no significant difference between FJ91 and FJ91-LUX (*t*-test)

To generate a LUX-tagged *R. solanacearum* strain, we transformed a plasmid containing the *lux*CDABE operon into *R. solanacearum* FJ91, and the transformed strain is hereafter referred to as FJ91-LUX. *R. solanacearum* FJ91 was isolated from Fujian province of China. Clear bioluminescent signals were observed in the FJ91-LUX colonies under a photon camera (Photek HRPCS5), while no signals were detected in FJ91 under the same conditions (Fig. 1a). The shape of FJ91-LUX colonies was not significantly different from that of FJ91. The colonies showed a red-colored smooth circular shape on solid media containing triphenyl tetrazolium chloride (TTC), which was converted to red insoluble formazan products by bacterial dehydrogenases (Fig. 1a). To rule out the possibility that *lux*CDABE expression might alter the biological characteristics of *R. solanacearum*, we first examined the growth curve of FJ91-LUX. We found that, similar to the control (FJ91), FJ91-LUX started the logarithmic growth phase 7 h after culturing and reached a stable phase at approximately 22 h (Fig. 1b). Formation of biofilms through secretion of extracellular polysaccharides and other substances that adhere to the contact surface is an important mechanism for bacteria to adapt to the environment [25]. Therefore, we next compared the biofilms of FJ91-LUX and FJ91; however, no significant difference was observed between the two biofilms (Fig. 1c). Motility is also an indicator of bacterial growth and pathogenicity [26]. Therefore, we evaluated the motility of FJ91-LUX and found that it displayed the same motility as FJ91 (Fig. 1d). Collectively, these results suggest that the expression of the *lux*CDABE operon does not alter the biological characteristics of *R. solanacearum*.

### Light intensity of FJ91-LUX is linearly related to the bacterial concentration

**Fig. 2:**
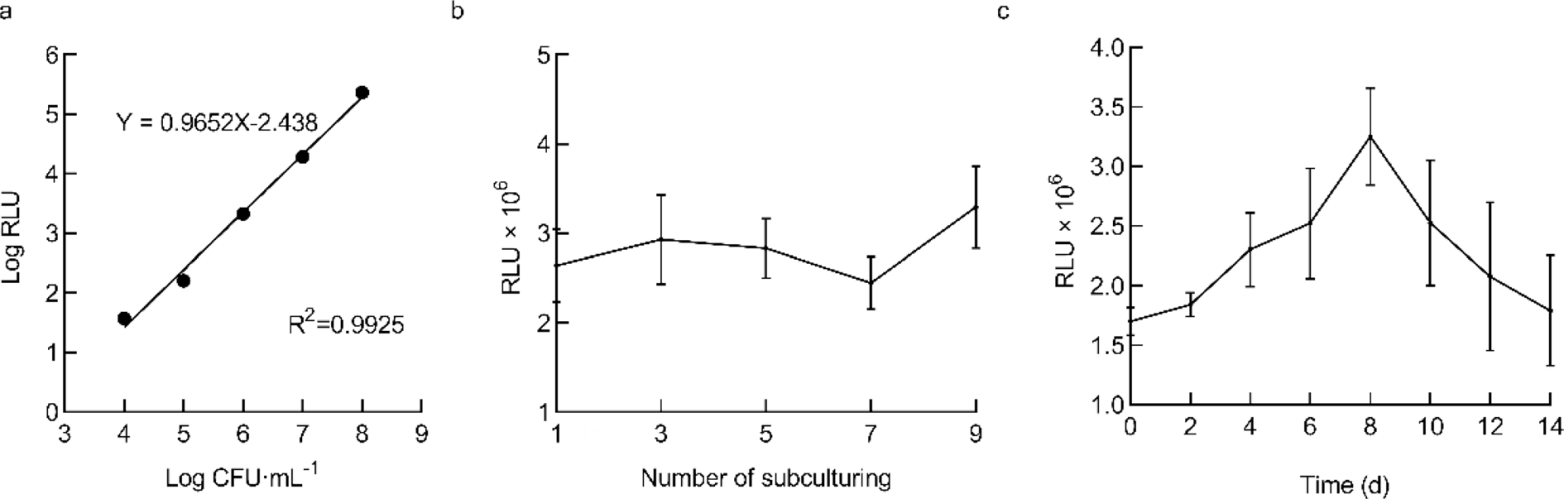
The light intensity of *Ralstonia solanacearum* FJ91-LUX is linearly correlated to its concentration. **a** Regression analysis of bioluminescent signals versus bacterial concentrations. The bioluminescent signals of the bacteria at the indicated concentrations were measured. Data are shown as the mean ± SD (n = 3). **b** Bioluminescent signals of bacteria after successive subculturing. Data are shown as the mean ± SD (n = 3). **c** Light intensity of FJ91-LUX after continuous growth on solid media. Data are shown as the mean ± SD (n = 4)

To investigate whether the bioluminescence of FJ91-LUX is related to its concentration, we measured the light signals at different bacterial concentrations (10^4^, 10^5^, 10^6^, 10^7^, and 10^8^ CFU·mL^−1^) (Fig. 2a). We found that signal intensity had a linear logarithmic relationship with bacterial concentrations within the range of 10^4^ to 10^8^ CFU·mL^−1^ (Fig. 2a), and the correlation coefficient was R^2^ = 0.9925. Together, these results suggest that the light signals emitted from FJ91-LUX are a reflection of bacterial concentration.

Spontaneous loss of plasmid might be a potential problem during bacterial subculturing; therefore, we measured the light intensity for 9 generations of successive subculturing. We found that the light intensity of FJ91-LUX was not significantly reduced after subculturing (Fig. 2b). However, when FJ91-LUX was grown continuously on solid media for 14 d, its signals gradually increased until 8 d, and then decreased to basal levels at 14 d after culturing (Fig. 2c), probably because of the aging of bacteria. These results suggest that the aging of bacteria might reduce the light signals of FJ91-LUX; therefore, it is advisable to measure the signals in the early phase after culturing.

### Real-time monitoring of the infection progress of FJ91-LUX in tomato

**Fig. 3:**
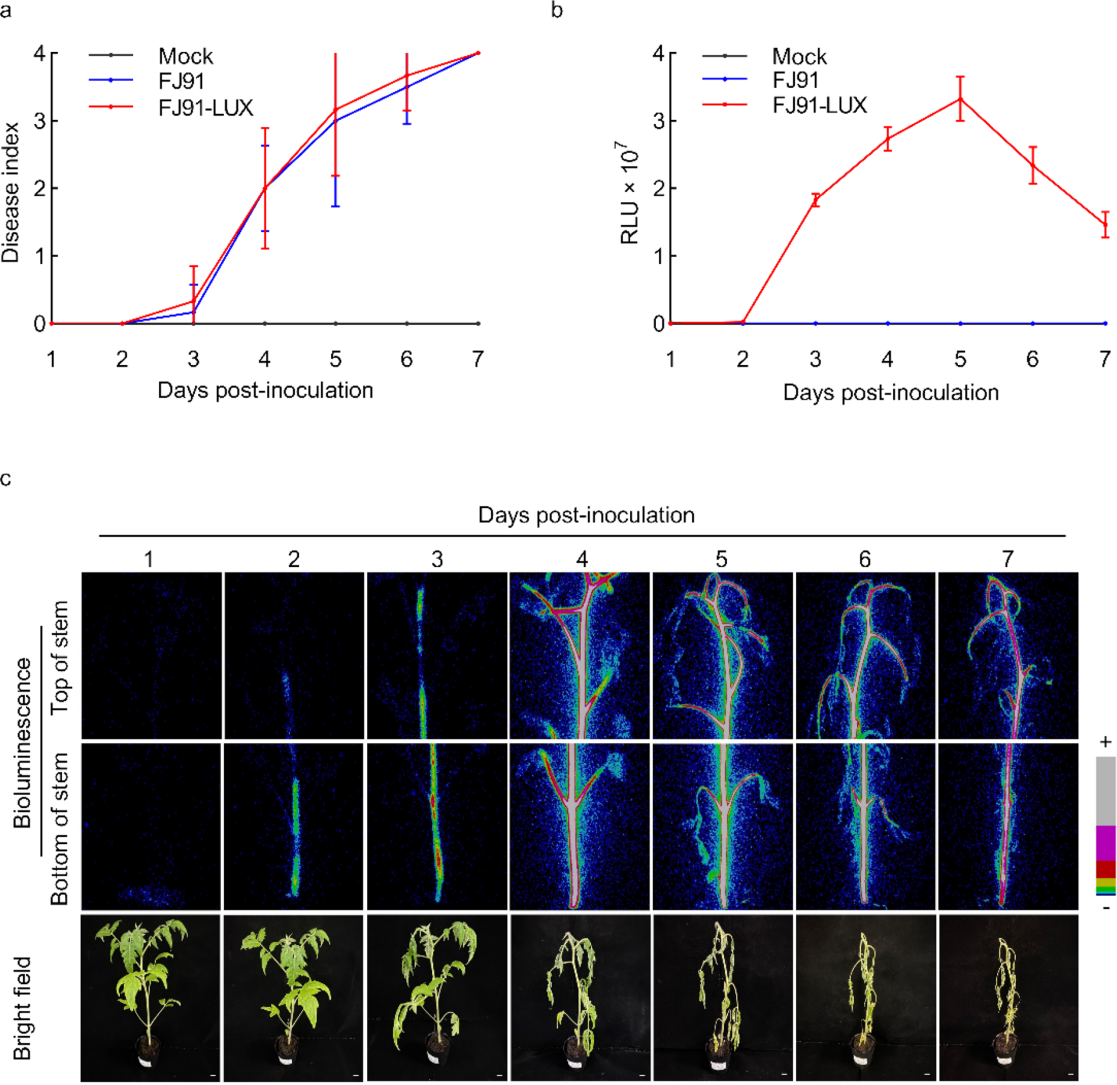
Real-time monitoring of the infection progress of *Ralstonia solanacearum* FJ91-LUX in tomato. **a** Pathogenicity of *R. solanacearum* FJ91-LUX in tomato was not significantly different from that of FJ91. The disease index of tomato plants was scored after inoculation at the indicated time points. Disease index ranges from 0 to 4: 0 (no wilting), 1 (1–25% wilted), 2 (26–50% wilted), 3 (51–75% wilted), and 4 (76–100% wilted or dead). Data are shown as the mean ± SD (n = 6). **b** Light intensity of tomato plants after inoculation. Data are shown as the mean ± SD (n = 6). **c** Bioluminescent images of representative tomato plants. + : Strong; −: Weak. Bar = 1 cm

To evaluate whether the expression of *lux*CDABE affects the pathogenicity of *R. solanacearum*, we compared the disease index of tomato after inoculation with FJ91-LUX and FJ91. Tomato seedlings (28-d-old) were soil-soak inoculated with bacteria (OD_600_ = 1), and the disease index was scored every day after inoculation. The disease index was empirically categorized into five grades: 0 grade (no leaf wilting was observed), 1st grade (25% of the whole leaves were wilted), 2nd grade (50% of the whole leaves were wilted), 3rd grade (75% of the whole leaves were wilted), and 4th grade (100% of leaves were wilted or the entire plant died). Tomato seedlings started to wilt 3 d post inoculation (dpi), and the disease index reached 2nd grade at 4 dpi. More severe wilt symptoms were observed at 5 dpi, and all plants died by 7 dpi (Fig. 3a, b). The disease index after inoculation with FJ91-LUX was not significantly different from that of FJ91 (Fig. 3a). These results suggest that *lux*CDABE gene expression does not alter the pathogenicity of *R. solanacearum*.

We next determined whether FJ91-LUX could be used to monitor the infection progress of *R. solanacearum* in tomato. We measured the light signals of tomato seedlings every day after inoculation with FJ91-LUX using a photon camera. We found that a weak signal was detected at the base of the tomato stem at 2 dpi, suggesting that it took one or two days for the bacteria to move from the root to the stem (Fig. 3c). Light signals were observed on the upper part of the stem at 3 dpi and continuously increased to reach maximal levels at 5 dpi. It has been reported that *R. solanacearum* mainly infects plant stems [27]. In agreement with these results, we observed light signals mainly in the stem region and rarely in the leaves (Fig. 3c). Even in the dying plants at 6–7 dpi, *R. solanacearum* did not seem to infect tomato leaf mesophyll cells. It is worth noting that fewer signals were detected at 6–7 dpi than at 5 dpi, indicating that the bacteria might lose their viability in the dying plants, consistent with the observation that aging bacteria reduced the light signals (Fig. 2c). Collectively, FJ91-LUX can be used for real-time monitoring of bacterial infection in tomato.

### Real-time monitoring of the infection progress of FJ91-LUX in Arabidopsis

**Fig. 4:**
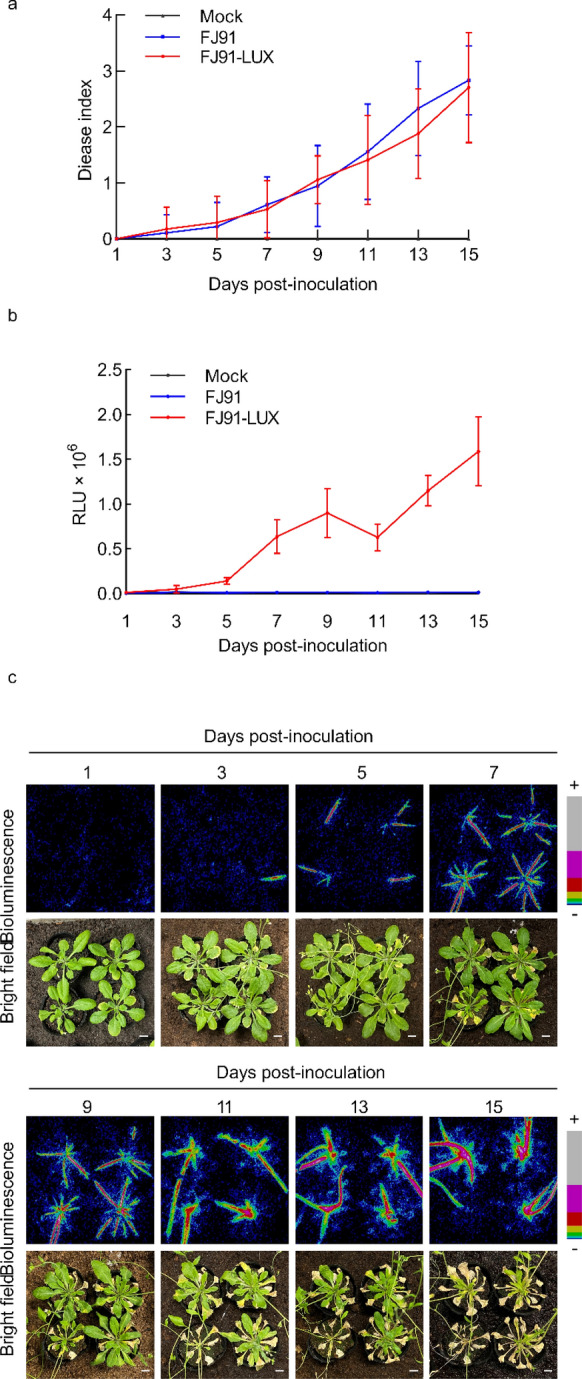
Real-time monitoring of the infection progress of *Ralstonia solanacearum* FJ91-LUX in Arabidopsis. **a** Pathogenicity of *R. solanacearum* FJ91-LUX in Arabidopsis is not significantly different from that of FJ91. *R. solanacearum*-inoculated Arabidopsis was scored every other day using a disease index ranging from 0 to 4: 0 (no wilting), 1 (1–25% wilted), 2 (26–50% wilted), 3 (51–75% wilted), and 4 (76–100% wilted or dead). Data are shown as the mean ± SD (n = 6). **b** Light intensity of Arabidopsis plants after inoculation. Data are shown as the mean ± SD (n = 6). **c** Bioluminescent images of representative Arabidopsis plants. + : Strong; −: Weak. Bar = 1 cm

We next determined whether FJ91-LUX could be used to monitor the progression of *R. solanacearum* infection in Arabidopsis. Arabidopsis mature plants (24-d-old) were soil-soak inoculated with FJ91-LUX and FJ91 (OD_600_ = 1), and light signals were monitored every two days after inoculation. Consistent with the results in tomato, it was observed that FJ91-LUX exhibited the same level of pathogenicity in Arabidopsis, as did the strain FJ91 (Fig. 4a). Arabidopsis plants wilted and died at 15 dpi in cases of infection by either of the two strains of *R. solanacearum* (Fig. 4a). No light signal was detected at 1 dpi with FJ91-LUX, a weak signal was observed at 3 dpi, and then the light signals gradually increased from 3 to 9 dpi (Fig. 4b, c). The light signals were mainly found in the vein of rosette leaves before 9 dpi, and moved from rosette leaves to stems at 11 dpi, without diffusing in the mesophyll cells of rosette leaves (Fig. 4c). Compared with the light intensity at 9 dpi, the levels at 11 dpi were slightly decreased, probably because the rosette leaves withered at 11 dpi, leading to the reduced total signals. However, the signals in stems increased from 11 to 15 dpi (Fig. 4b, c). Collectively, our results suggest that FJ91-LUX can be used for real-time monitoring of bacterial infection progress in Arabidopsis.

**Fig. 5:**
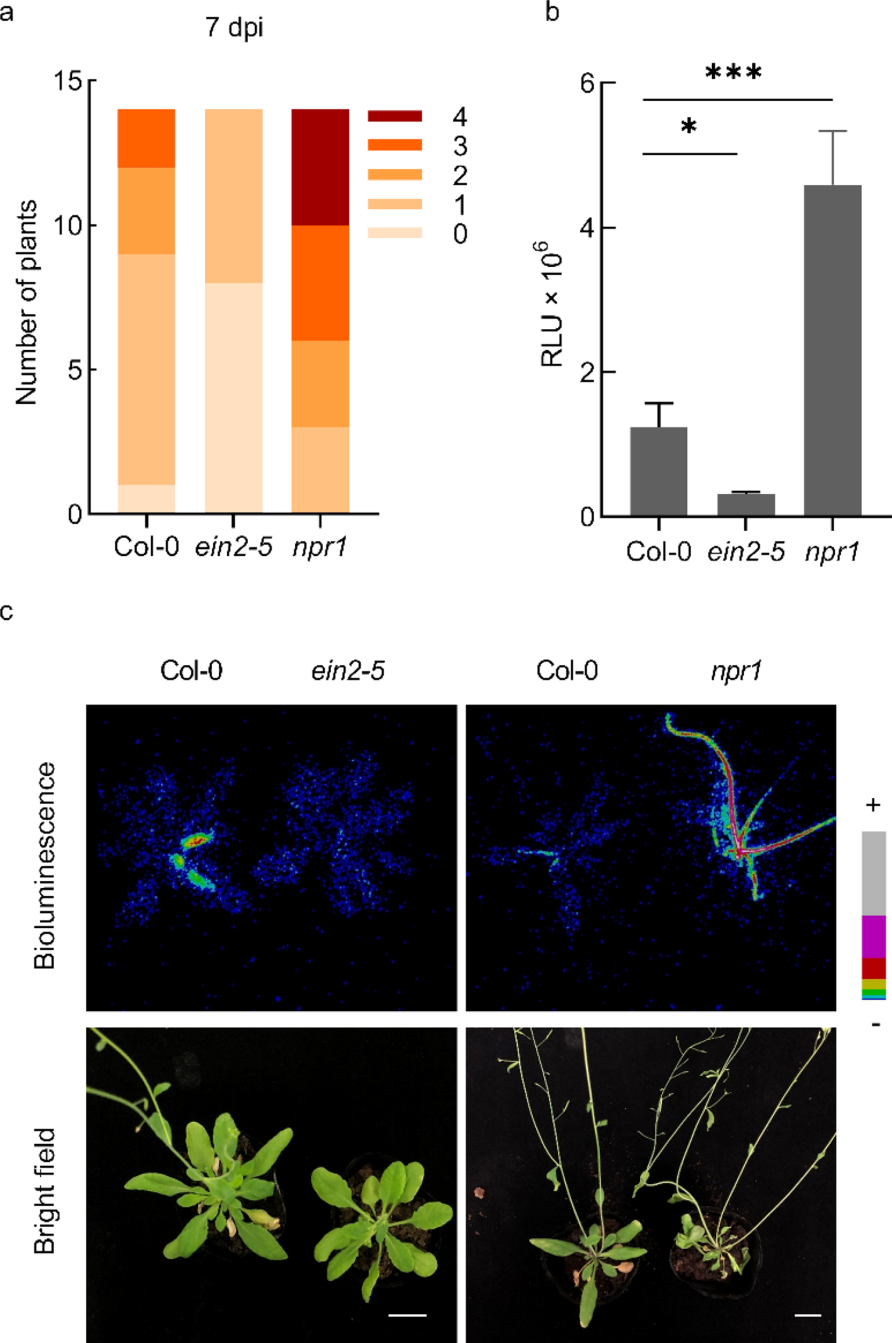
Light intensity of *ein2* and *npr1* mutants after inoculation with *Ralstonia solanacearum* FJ91-LUX. **a** Disease index of Col-0, *ein2-5*, and *npr1* mutants inoculated with FJ91-LUX. *R. solanacearum*-inoculated Arabidopsis were scored 7 d post inoculation (dpi). Disease index: 0 (no wilting), 1 (1–25% wilted), 2 (26–50% wilted), 3 (51–75% wilted), and 4 (76–100% wilted or dead). **b** Bioluminescent signals of Col-0, *ein2-5*, and *npr1* mutants inoculated with FJ91-LUX. Data are shown as the mean ± SE (n = 14, *P ≤ 0.05, ***P ≤ 0.001, *t*-test). **c** Bioluminescent images of representative plants inoculated with FJ91-LUX. + : Strong; −: Weak. Scale = 1 cm

### Quantification of plant resistance to *R. solanacearum* using FJ91-LUX

The light signals of FJ91-LUX were linearly correlated to their concentrations, when the infected plants were alive. We next examined whether FJ91-LUX could be used for the quantification of plant resistance to *R. solanacearum*. It has been reported that Arabidopsis *ETHYLENE INSENSITIVE 2* (*EIN2*) gene plays a negative role in resistance to *R. solanacearum*, and *ein2* mutants show delayed wilt symptoms [12]. Therefore, we inoculated Arabidopsis wild-type (Col-0) plants and *ein2-5* mutants with FJ91-LUX and compared the light intensity at 7 dpi. Consistently, we found that *ein2-5* mutants showed reduced disease index (Fig. 5a), and the light intensity in *ein2-5* mutants was significantly lower than that in the wild type (Fig. 5b, c). In contrast, mutants with defects in *NON EXPRESSER OF PATHOGENESIS RELATED 1* (*NPR1*, encoding a key transcriptional co-activator in SA signaling pathway) exhibited significantly stronger light intensity than wild type after inoculation with FJ91-LUX. Together, these results suggest that FJ91-LUX could be used for the quantification of plant resistance to *R. solanacearum* and possibly facilitate large-scale mutant screens in the future.

## Discussion

Bacterial wilt, caused by *R. solanacearum*, is one of the most devastating bacterial diseases worldwide. Owing to the limitations of the available methods, a fast real-time disease detection method for Arabidopsis is required. In this study, we generated a *luxCDABE-*labeled *R. solanacearum*, which allowed the use of bioluminescence imaging technology to detect bacterial wilt disease in tomato and Arabidopsis.

Numerous methods have been developed to determine plant disease resistance, such as disease index, bacterial growth by colony counting, detection of bacterial concentration by PCR or loop-mediated isothermal amplification (LAMP), serological assays (e.g. ELISA, enzyme-linked immunosorbent assay), hyperspectral imaging by remote sensors, or bioluminescence imaging technology [14, 15, 28]. Each method has its own advantages and potential pitfalls, and bioluminescence imaging technology provides the biggest advantage in terms of providing noninvasive, spatial, quantitative, and real-time monitoring of the infection progress [17]. This technology will provide a powerful tool for the study of disease-resistant mechanisms, as well as the breeding of disease-resistant varieties.

Bioluminescent bacterium can be generated by transforming a plasmid carrying the *lux*CDABE operon or inserting the *lux*CDABE operon directly into the genome of the bacterium [29, 30]. Expression of *lux*CDABE in plasmids is usually higher compared to its expression when inserted into the genome; therefore, bacteria carrying plasmid-expressed *lux*CDABE display stronger light signals and are easily detected. Although the loss of plasmids during successive subculturing is a potential pitfall, we found that the light signals of FJ91-LUX persisted even after 9 generations of subculturing. However, we cannot rule out the possibility of the loss of plasmid during infection in plant tissues. Therefore, insertion of the *lux*CDABE operon into the genome is an alternative method. To increase the expression of the *lux*CDABE core gene in the genome, other LUX components may be added; for example, luxF and luxG enhance the luminescence intensity of bacteria [31, 32]. Improving the luminous intensity of luxCDABE can further improve the sensitivity of the system.

The *lux*CDABE-tagged *P. syringae* strain has been used for large-scale screening of resistant ecotypes in Arabidopsis [20]. Here, we generated a *lux*CDABE-tagged *R. solanacearum*, which could also be used for large-scale screening of Arabidopsis mutants or ecotypes with altered resistance to *R. solanacearum* infection. Compared with wild type, *ein2-5* and *npr1* mutants showed altered light intensity and wilt symptom after inoculation with *lux*CDABE-tagged *R. solanacearum*. However, to date, the role of ethylene and SA signaling pathway in resistance to *R. solanacearum* remains poorly understood. Remarkably, we found that, in Arabidopsis, the light intensity in the stems at 15 dpi was 2 folds higher than that in rosette leaves at 9 dpi (Fig. 4c), suggesting that the developmental stage is critical for *R. solanacearum* proliferation. Therefore, the low light intensity in *ein2-5* mutant might be due to its delayed bolting (Fig. 5c). In the case of SA signaling pathway, several lines of evidence indicate that NPR1 plays a positive role in resistance to *R. solanacearum* [33, 34], on the other hand, the Arabidopsis SA-deficient mutant *sid2* (encoding isochorismate synthase) and *NahG* (encoding salicylate hydroxylase) transgenic lines do not display significant increase in bacterial proliferation [7, 13]. Therefore, function of ethylene and SA in resistance to *R. solanacearum* requires further analysis.

It is worth noting that the light intensity in tomato was reduced at the later stage because of bacterial death (Fig. 3c); therefore, it cannot reflect the disease severity at this stage for tomato. To avoid this, it is advisable to quantify the light signals at a relatively early stage after inoculation, or to monitor the kinetics first, to select the bacterial growth stage. On the other hand, this suggests that the *lux*CDABE operon provides a means of detecting bacterial viability and can be used to monitor chemical toxicity [29, 35]. Therefore, it is plausible that FJ91-LUX could be used in the future to facilitate the testing of bacterial antibiotics, DNA and membrane damage, or oxidative stress chemicals.

## Conclusions

In this study, we generated a *luxCDABE-*labeled *R. solanacearum* strain that allows tracking of the bacterial movement and quick quantification of the disease intensity in a living host.

## Methods

### Bacterial strains and growth conditions

*R. solanacearum* FJ91 strain (CGMCC No. 1.12711) was isolated from Fujian Province, China [36]. *R. solanacearum* were cultivated in casamino acid-peptone-glucose (CPG) media containing TTC [37]. *P. syringae* and *Escherichia coli* DH5α strains were grown in Luria–Bertani medium. Bacterial concentrations were determined by measuring the absorbance at OD_600_.

### Plasmid construction

A promoter-less *lux*CDABE operon was amplified from a *P. syringae*-LUX strain [20] by PCR using the primers *lux*F (5-CCGGAATTCATGACTAAAAAAATTTCATTCAT-3) and *lux*R (5-CGCGGATCCATCAACTATCAAACGCTTC-3), and ligated to the pBBR1MCS2 plasmid to generate pBBR1MCS2- *lux*CDABE plasmid. The inserted fragment was verified by sequencing.

### Generation of FJ91-LUX strain

FJ91-LUX was generated by transforming the pBBR1MCS2-*lux*CDABE plasmid into *R. solanacearum* FJ91. Briefly, freshly grown *R. solanacearum* colonies were cultured in 5 mL of CPG medium at 28 °C for 16 h with constant shaking at 200 rpm. Aliquots (1 mL) were centrifuged at 3500×*g* at 4 °C for 5 min and resuspended in 300 mM sucrose solution. The cell pellet was washed twice and dissolved in 100 μL of 300 mM sucrose solution. The pBBR1MCS2-*lux*CDABE plasmid was then transformed into *R. solanacearum* competent cells by electroporation (V = 2.2 kV·cm^−1^, C = 25 μF, R = 400 Ω) to generate FJ91-LUX.

### Bioluminescence assay

Bioluminescent signals were monitored using a photon camera, version HRPCS5 (Photek Ltd., UK). Images and data were processed using Image32 software, a program coupled with a photon camera. The major parameters of the camera setup were the binary slice and 10% ND filter. The photon counts were integrated over 5 min.

### Growth curve determination

Bacteria in liquid media were collected after overnight cultures by centrifugation at 3000×*g* for 5 min, and the pellet was resuspended in sterile water. After washing three times with sterile water, the solution was adjusted to an OD_600_ of 0.005 using fresh liquid medium. The OD_600_ was measured every 0.5 h.

### Biofilm assay

Bacterial biofilm formation was measured by the crystal violet staining method [38]. In brief, 100 μL of the bacterial culture (OD_600_ = 0.5) was cultivated in a 96-well cell culture plate for 72 h. The medium was removed, followed by gentle washing (three times) with 200 µL of sterile water. Then, 125 μL of 0.1% (w/v) crystal violet (Aladdin, China) solution was added for 30 min. The dye solution was discarded and the plates were dried at 24 °C, and the bound stain was dissolved in 1% sodium dodecyl sulfate. The absorbance was measured at 570 nm using a Multiskan FC microplate spectrophotometer (Thermo Fisher Scientific, USA).

### Mobility measurement

The mobility assay was performed as described previously [39]. In brief, one drop of bacterial culture (5 μL, OD600 = 0.6) was placed at the center of a CPG plate with 0.3% agar and incubated at 28 °C after drying in a laminar airflow hood. The colony diameter was measured after 2 days.

### Subculturing in liquid media

The bacteria were initially grown in liquid media to OD_600_ = 1, and then diluted 1000 folds with fresh media to continue growth. This passaging procedure was repeated 9 times, and bioluminescent signals were measured every time before dilution.

### Plant materials and growth conditions

The seeds of Arabidopsis *ein2-5* [40], *npr1* [41] mutants and wild type Col-0 were sterilized with 10% bleach for 10 min, rinsed with sterile water three times, and grown on 1/2 Murashige-Skoog agar plates. Seedlings were transferred to a 6 × 6 cm pot containing peat-based compost (Sun Gro Horticulture, USA), and maintained in a growth chamber (Conviron, Canada) under conditions of 16 h photoperiod, 75% humidity, and 22 °C temperature. Tomato (*Solanum lycopersicum*) ecotype Zheza 809 (Zhejiang Academy of Agricultural Sciences, China) was used in this study. Seed sterilization was the same as that of Arabidopsis, except that it was treated with bleach for 20 min. Tomato plants were grown in a growth room under conditions of 16 h photoperiod, 75% humidity, and 25 °C temperature.

### Inoculation of *R. solanacearum*

Plants were inoculated with *R. solanacearum* using a root-soaked approach [42, 43]. Bacterial overnight cultures were collected by centrifugation and adjusted to an OD_600_ of 0.1. For tomato inoculation, roots of four-week-old tomato seedlings were scratched with a blade at 1 cm from the hypocotyl, and then 50 mL of bacterial suspension was drenched around the root zone. For Arabidopsis inoculation, the pot with four-week-old Arabidopsis plants was cut off half from the bottom and the upper half was immediately submerged into a bacterial suspension. After incubation with bacteria for 20 min, the pots were carefully moved to a new tray with a thick layer of moist soil and covered with a dome to keep the moisture for 2 d.

### Statistical analysis

Statistical analyses and data normality tests were performed using Excel, SPSS 20.0, and all graphs were generated using GraphPad Prism 8.

## Data Availability

All data generated or analysed during this study are included in this published article.

## Abbreviations

CPG: Casamino peptone agar
TTC: Triphenyl tetrazolium chloride
